# Yellow Fever and the Mosquito

**Published:** 1902-01

**Authors:** 


### TEXAS MEDICAL JOURNAL.

AUSTIN, TEXAS.

A MONTHLY JOURNAL OF MEDICINE AND SURGERY.

EDITED AND PUBLISHED BY
F. E. DANIEL, M. D.

ASSOCIATE EDITOR:
WITTEN BOOTH RUSS, M. D.,
San Antonio, Texas.

Published Monthly at Austin, Texas. Subscription price $1.00 a year in advance.

Eastern Representative: John Guy Monihan, St. Paul Building, 220 Broadway, New York City.

Official organ of the State Association of Health Officers, the West Texas Medical Association, the Houston District Medical Association, the Austin District Medical Society, the Brazos Valley Medical Association, the Galveston County Medical Society, and several others.

### YELLOW FEVER AND THE MOSQUITO.

Dr. Oliver's paper in this issue of the JOURNAL makes a strong case against the postulate that the mosquito is the sole cause of the spread of yellow fever. I hope every reader of the JOURNAL will read it.

The evidence that yellow fever may be, and is, disseminated by *fomites* is overwhelming. Granting (which I do not) that the experiments of Drs. Reed, Carroll and Agramonte in Cuba have demonstrated sufficiently that the mosquito is one factor in propagating the disease, it does not follow by any means that there are not others. Indeed, my personal experience of yellow fever has convinced me that the disease is introduced into a town either by goods or merchandise or some such carriers of infection, packed in bales, boxes or trunks; or by a person in whom the disease is incubating and develops after arrival; that each case of the disease and every corpse is a focus from which the infection radiates; and while the fever is not contagious, in the sense that we understand measles and scarlet fever to be contagious, for instance, it is highly *infectious;* that is, a nonimmune breathes the infected atmosphere in a close room, and, even, when an epidemic is prevailing, the atmosphere of a town or city—and acquires the disease. No man has ever seen the "germ" or whatever the cause of yellow fever may be; and no one knows what it is. If it were a bacillus it would have been discovered long ago. It is probably a *spore,* and is ultra-microscopic, and develops into something else after having entered the blood through the respiratory organs. That it attacks, and in fatal cases decomposes the blood is well established.

As Dr. Oliver has cited the records and shown very clearly that the disease is spread by persons with the disease, and by *fomites* in various forms, and that there is no interval of twelve days between the first case occurring in a community and subsequent cases, as is claimed by those who advocate the mosquito theory, I will have to discuss the subject from the standpoint of my personal experience. A fatal defect in the mosquito theory is, it cannot be shown where the mosquito gets his poison with which to inoculate *the first case.* How does a first case occur in a town where the patient has not been out of town, and is as remote, say, as Jackson is from Vicksburg (1878) from any cases or any infected city; or as Grenada or Holly Springs is from New Orleans; or as Edwards, Miss., is from Ocean Springs? The case in Jackson, cited by me in the December issue of the JOURNAL, had not been out of Jackson, nor had he been exposed to any infection other than that of the demijohn of brandy packed in sawdust. It is not probable that a *culex faciata* flew from Vicksburg to Jackson,—charged with a twelve day load of poison, and bit him. There was no twelve days interval between the occurrence of this case and the next,—the doctor who attended him, Dr. Harrington. Sneed (see December number) went from Vicksburg (infected to some extent; there had occurred one or two cases in Vicksburg then) to Lake, about one hundred miles east of Vicksburg in the piney woods, with his wife, children and baggage. The disease was then incubating in him, and on his arrival he went to bed in the back part of Saunder's store, sending his family and baggage to Col. Yarboro's, his father-in law, up in the residence part of the village. Sneed was in bed only a few days, suffering, as his physician, Dr. McCallum, said, with "a light malarial attack." In ten days or less, every person who had been in Sneed's room, including the negro who waited on him, and his physician, Dr. McCallum, was *dead.* They died rapidly, throwing up black vomit on the second or third day. One of the earliest cases resulting from this exposure was the druggist, Mr. McFadden, I think his name was. He lived in the suburbs, north of the town and very near the graveyard. He died, but his family, consisting of wife, three children and his father-in-law and mother-in-law—Mr. and Mrs. Saunders,—remained well some two weeks or more. I roomed in the house with them. There had been about thirty corpses interred in the little graveyard by the time of the occurrence I am about to relate. There came a heavy rainstorm one hot night in September (1878), and the graves, which were shallow and in light sand and gravel soil, were beat upon until the corpses were exposed. Such a stench arose from them that the buzzards hovered near. Within forty-eight hours every member of the household just across the road from the graveyard, the family of Mr. McFaddin and the Saunders, who, I forgot to state, had fled from the infected village to this suburban place of their son-in-law and had escaped, were taken in rapid succession, and every one perished, and perished rapidly, throwing up black vomit within ten or twelve hours after being attacked. There is record of this somewhere in Washington—in the report made to Congress after the epidemic. The late Dr. S. M. Bemis, of New Orleans, was a member of the commission to investigate and report upon the epidemic. He came to Jackson, where I was then living, and I went out to Lake with him and went over the ground, he taking notes of this and other occurrences.

It would seem scarcely worth while to cite evidence in support of what has been so well established as that yellow fever is disseminated by *fomites* and by atmospheric infection, were it not that the surgeon general of the United States army, one of the ablest bacteriologists in America, and one of the earliest investigators of yellow fever, and the surgeon general of the Marine Hospital Service, are both thoroughly and fully committed to the mosquito fad. In a paper by the former, Surgeon General Sternberg—published in the 1901 volume of reports of the Smithsonian Institution, Dr. Sternberg says:

"When a case is imported to one of our Southern seaport cities from Havana, Vera Cruz or some other endemic focus of disease, an interval of two weeks or more occurs before secondary cases are developed as a result of importation. In light of our present knowledge this is readily understood. A certain number of mosquitos, having filled themselves with blood from this first case, after an interval of twelve days or more, bite nonimmune individuals in the vicinity, and these individuals, after a brief period of incubation, fall sick with the disease. * * *."

Why twelve days? Do not mosquitos feed, or bite or suck, oftener than twelve days? Biting a sick man in New Orleans or

Vicksburg, do they fly to Jackson, Lake, Grenada or Holly Springs, holding their charge of poison purposely?

Dr. Sternberg says further: "It is evident that in view of our present knowledge relating to the mode of transmission of yellow fever, the preventive measures which have hitherto been considered most important, *i. e.*, isolation of the sick, disinfection of clothing and bedding and *municipal sanitation* [italics ours] are either of no avail, or of comparatively little value. It is true that yellow fever epidemics have resulted, as a rule, from the introduction to a previously healthy locality, of one or more persons suffering from the disease. But we now know that its extension did not depend upon the direct contact of the sick with non-immune individuals, and that isolation of the sick from such contact is unnecessary and without avail."

Here are assertions from the highest authority in America which directly and distinctly contradict the experience of every yellow fever physician in the South, and give the lie to the record of the disease from the earliest observations. Certainly it contradicts the history of the disease at Jackson, Lake, Holly Springs, Grenada, Edwards and other Mississippi towns in 1878 and 1897-8, and throughout Texas along the line of the Texas Central railroad in 1867, and at Columbus and other towns in Texas in 1873.

As a logical sequence, or a corollary of this mosquito theory,—for as yet it is only a theory—and not even a plausible one at that, Sternberg and Wyman to the contrary notwithstanding, it is seriously proposed to abolish quarantine! The Texas people are wedded to quarantine, and if the experiment is tried in this State it will result in a revival of the shotgun methods.

